# Bacteriophages as antimicrobial agents against bacterial contaminants in yeast fermentation processes

**DOI:** 10.1186/s13068-014-0123-9

**Published:** 2014-08-20

**Authors:** Juliano Bertozzi Silva, Dominic Sauvageau

**Affiliations:** Department of Chemical and Materials Engineering, University of Alberta, Electrical & Computer Engineering Research Facility (ECERF), 7th Floor, 9107-116 Street NW, Edmonton, Alberta T6G 2V4 Canada

**Keywords:** Bacteriophage, Yeast, Lactic acid bacteria, Contamination abatement, Ethanol, Biofuel, Antibacterial

## Abstract

**Background:**

The emergence of biofuels produced through yeast fermentation represents an important avenue for sustainable energy production. Despite all its advantages, this process is vulnerable to contamination by other organisms – most commonly lactic acid bacteria – that are present in raw feedstocks and/or in production lines. These contaminants compete with the yeast for nutrients, reducing the overall biofuel yield, and release substances that inhibit yeast growth. Here, we investigated the application of bacteriophages as potential antibacterial agents in yeast fermentation.

**Results:**

Experiments conducted to understand the impact of pH on yeast, bacterial, and phage development showed that the yeast *Saccharomyces cerevisiae* Superstart™ grew in a similar fashion at pH levels ranging from 3 to 6. Growth of *Lactobacillus plantarum* ATCC® 8014™ was inhibited by pH levels of less than 4, and phages ATCC® 8014-B1™ (phage B1) and ATCC® 8014-B2™ (phage B2) displayed different infectivities within the pH range tested (pH 3.5 to 7). Phage B1 showed the best infectivity at pH 6, while phage B2 was most virulent at pH levels ranging from 4 to 5, and the cocktail of these phages showed highest infectivity in the range from pH 4 to 6. Population dynamics studies in MRS medium at pH 6 showed that, in the presence of bacteria inoculated at 10^7^ cells/ml, yeast cultures were impeded under aerobic and anaerobic conditions, showing major decreases in both cell yield and ethanol production. The addition of the phage cocktail at a low initial multiplicity of infection was sufficient to reduce contamination by over 99%, and to allow yeast and ethanol yields to reach values equivalent to those of axenic cultures.

**Conclusions:**

From the results observed, phages are good candidates as antimicrobial agents, to be used in place of or in conjunction with antibiotics, in yeast fermentation processes. Their implementation with other common contamination abatement/prevention methods could further increase their efficacy.

## Background

Yeast fermentation has been extensively used by humans for millennia to intentionally synthesize various useful products such as foods, beverages, and chemicals [1,2]. In recent decades, there has been much research into the application of yeasts for the production of biofuels, with the emphasis on ethanol. Biofuels are considered an important player in the energy field because of their environmental appeal, particularly their production from renewable sources [3,4].

A common impediment to many yeast fermentations is the presence of undesirable contaminant microorganisms, mainly bacteria and wild yeasts [5]. The presence of such undesirable organisms in fermentations is a consequence of their natural occurrence in the agricultural raw materials that compose many mash feedstocks, and their prevalence in industrial facilities [6,7]. These contaminants compete for nutrients with the fermenting yeasts, and may release substances, such as organic acids, that can inhibit yeast growth [8]. Consequently, the fermenting yeasts do not thrive and lower ethanol yields are often obtained [9].

The majority of contaminants found in industrial plants are lactic acid bacteria (LAB) [10–14]. These are aero-tolerant anaerobic, Gram-positive bacteria that can be further divided into two groups – homofermenters and heterofermenters – depending on their products of glucose metabolism [15–17].

Several methods are used, often in conjunction, to eliminate or, at least, abate contaminants. These include heat treatment, cleaning and sanitation, reduction of the pH of the feedstock, and addition of antibiotics [7]. Each of these approaches has advantages and disadvantages. Antibiotics are advantageous for acting selectively against bacteria, without exhibiting detrimental effects on the fermenting yeasts. However, application of antibiotics, even for non-therapeutic purposes, is known to facilitate the emergence of resistant bacterial strains [18]. Concerns arising from increased bacterial antibiotic resistance include reduction in the effectiveness of the applied antibiotic, and public health issues caused by the presence of residual amounts of antibiotics in fermentation co-products used as feed for livestock (for example, distillers’ dried grains with solubles (DDGS)) [10,19]. As a result, regulations imposing antibiotic-free feedstocks are phasing out the usage of these antimicrobials, especially in Europe [7]. There is thus an increasing drive to find alternatives to antibiotics in yeast fermentation processes, and the number of studies in this area of research is also increasing [20–25].

In the present work, the application of strictly lytic bacteriophages (hearafter referred to as ‘phages’) was investigated for the reduction of bacterial contaminants in yeast fermentation processes. Phages are viruses whose hosts are bacteria and archaea [26]. They are ubiquitous, and compose the most numerous and diverse population of biological entities on Earth [27]. In addition, many are capable of rapidly replicating to high numbers at the expense of their host cell. The application of phages as antibacterial agents has mostly revolved around the replacement of antibiotics in therapeutic purposes (phage therapy) [28,29], but work has also been carried out to develop applications in non-therapeutic systems [30].

The concept of utilizing phages to control contamination in ethanol production has been briefly mentioned in the literature [7] and has been the subject of two patent applications [31,32]. However, to our knowledge, no systematic studies to establish a comprehensive understanding of their use in yeast fermentations have been reported thus far. In the present study, we aimed to establish preliminary parameters for the application of phages as a stand-alone or a complementary technology for the control and abatement of bacterial contamination in yeast fermentation processes.

## Results

### Impact of pH on growth and infection under aerobic conditions

**Figure 1:**
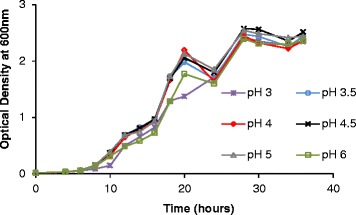
**Growth of** ***saccharomyces cerevisiae*** **under aerobic conditions.** Experiments conducted in M9 medium at initial pH levels ranging from pH 3 to pH 6. Values are averages of duplicate experiments.

**Figure 2:**
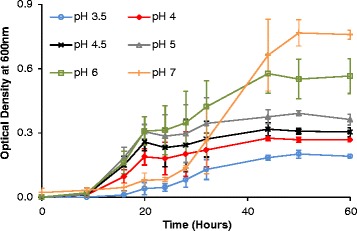
**Growth of** ***lactobacillus plantarum*** **under aerobic conditions.** Experiments conducted in M9 medium at initial pH levels ranging from pH 3.5 to pH 7. Error bars indicate the standard deviation of three experiments.

Growth and infection experiments were conducted with yeasts, bacteria, and phages in M9 medium at different initial pH levels to assess the behavior of the cultures and to define phage infectivity. Figures 1 and 2 show the growth of yeasts and bacteria, respectively, under different initial pH conditions. The yeast strain used for the study grew similarly under all conditions tested, but lower initial pH led to lower final bacterial counts (Figure 2). Also of note, a longer lag phase was observed for bacterial cultures growing at an initial pH of 7. This is because pH 7 is not optimal for the growth of this *Lactobacillus* species [33]. The initial slow growth of the culture produced organic acids that lowered the pH of the medium to an extent that allowed faster growth afterwards, and this was corroborated by the maximum growth rate being similar to that of other cultures. However, despite the longer lag phase, it should be noted that the final yield was highest when the initial pH was 7.

**Figure 3:**
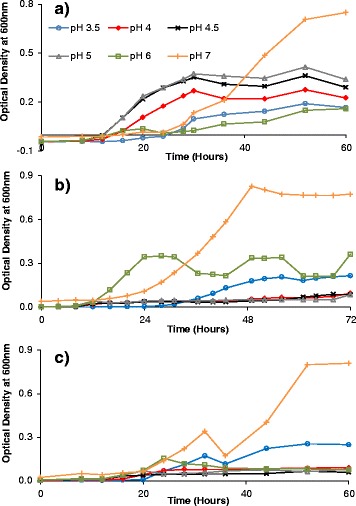
**Growth of** ***lactobacillus plantarum*** **infected by phages under aerobic conditions. (a)** Phage B1, **(b)** phage B2 and **(c)** cocktail of phages B1 and B2. Experiments were conducted at an initial multiplicity of infection (MOI) of 0.1 in M9 medium at initial pH levels ranging from pH 3.5 to pH 7.

**Figure 4:**
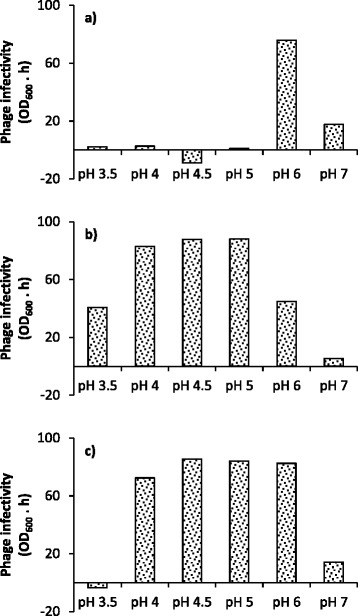
**Quantification of phage infectivity.** Infectivity was determined for M9 medium at initial pH levels ranging from pH 3.5 to pH 7. **(a)** Phage B1, **(b)** phage B2, and **(c)** cocktail of phages B1 and B2. Infectivity was calculated as the difference in the integrals of the curves in Figures 3 and 2.

Figure 3 demonstrates the growth of the bacterial host when infected by phage B1 (Figure 3a), phage B2 (3b), and the cocktail of phages B1 and B2 (3c) at different initial pH levels. Comparisons of these infected cultures with uninfected host cultures (Figure 2) at the same respective initial pH were conducted to assess phage infectivity. To this end, the integral of each curve in Figure 3 was calculated and subtracted from the integral of the curve of the corresponding uninfected culture (Figure 2). As these integrals indicate bacterial proliferation over the length of infection, the resulting differences in areas represent a quantitative measure of cell-time reduction caused by the phages. This, in turn, indicates how infective a phage is under the conditions tested. Phage infectivity for phages B1 and B2 alone and in combination is shown in Figure 4.

Figure 4 it can be observed that phage B1 had high infectivity at pH 6 (Figure 4a), phage B2 was most virulent from pH 4 to pH 5 (Figure 4b), and the infectivity of both phages combined was high and consistent in the pH range of 4 to 6 (Figure 4c). The slightly negative values of infectivity, observed for phage B1 at pH 4.5 (Figure 4a) and for the cocktail of phages at pH 3.5 (Figure 4c) (−8.9% and −3.6%, respectively) were a result of experimental error.

### Population dynamics of yeasts, bacteria, and phages B1 and B2

**Figure 5:**
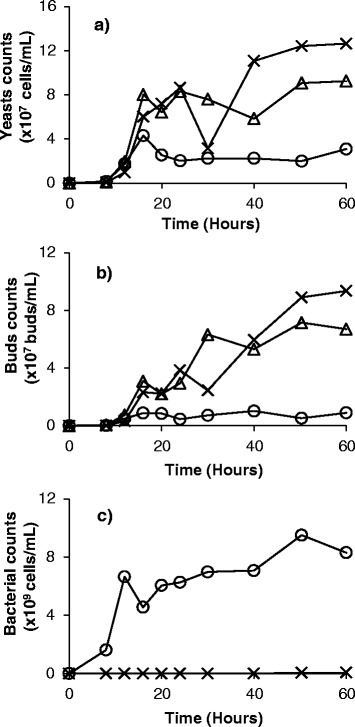
**Growth of yeast, bacteria, phage B1, and phage B2 under aerobic conditions**. Experiments conducted in MRS medium at pH 6. Counts of **(a)** yeast cells, **(b)** yeast buds, and **(c)** bacterial cells, measured using microscopy and a counting chamber, are presented. Data are reported for cases where yeasts were grown alone (Δ); in the presence of bacteria (O); and in the presence of bacteria and phages B1 and B2 (X). Values are averages of duplicates.

**Figure 6:**
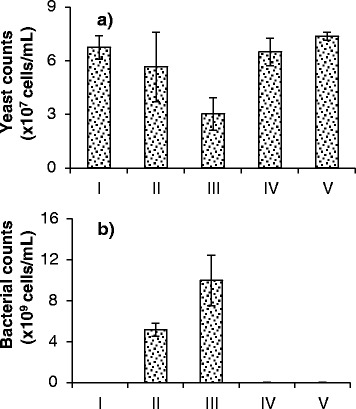
**Final cell counts for cultures of yeast, bacteria, phage B1, and phage B2 growing under aerobic conditions.** Experiments conducted in MRS medium at pH 6. Counts of **(a)** yeast and **(b)** bacterial cells, measured using microscopy and a counting chamber, are presented. Data are reported for cases where yeasts were grown alone (I); in the presence of bacteria at low inoculation level (II); in the presence of bacteria at high inoculation level (III); in the presence of bacteria at low inoculation level and phages B1 and B2 (IV); and in the presence of bacteria at high inoculation level and phages B1 and B2 (V). Measurements were taken at 20 hours of fermentation. Error bars indicate the standard deviation of three experiments.

**Figure 7:**
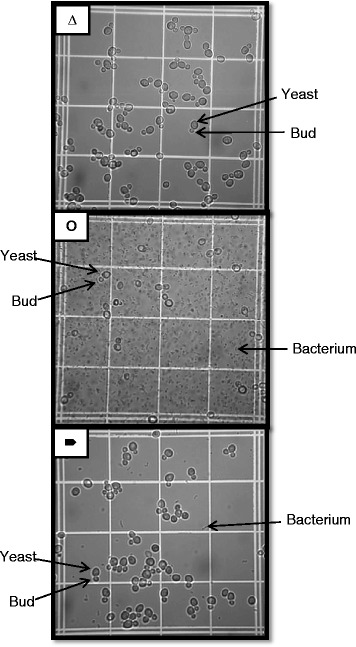
**Sample pictures for the population dynamics experiment presented in Figure** 6 **.** Data are reported for cases where yeasts were grown alone (Δ); in the presence of bacteria (O); and in the presence of bacteria and phages B1 and B2 (×). Examples of yeast cells, yeast buds, and bacterial cells are indicated in the pictures. All samples were diluted fivefold.

Figures 5 and 6 contain the results for population dynamics in MRS at pH 6 under aerobic conditions. These figures clearly show major reductions in yeast cell counts in the presence of bacteria (Figure 5a; Figure 6a, cases II and III). Additionally, significant reduction (over 99%) in bacterial cell counts was observed when phages were added (Figure 5c; Figure 6b, cases IV and V). The addition of phages allowed yeast cells to reach the same levels as axenic yeast cultures (Figure 5a; Figure 6a, cases I, IV and V). Sample pictures of cases I, III and V from Figure 6 can be seen in Figure 7, where the impact of the presence of phages on the yeast and bacterial populations can be observed.

**Figure 8:**
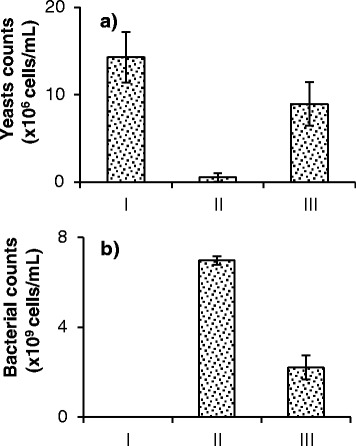
**Final cell counts for cultures of yeast, bacteria, phage B1, and phage B2 growing under anaerobic conditions.** Experiments conducted in MRS medium at pH 6. Counts of **(a)** yeast and **(b)** bacterial cells, measured using microscopy and a counting chamber, are presented. Data are reported for cases where yeasts were grown alone (I); in the presence of bacteria at high inoculation level (II); and in the presence of bacteria at high inoculation level and phages B1 and B2 (III). Measurements were taken after 65 hours of fermentation. Error bars indicate the standard deviation of three experiments.

Figure 8 shows results for yeast and bacterial population yields under anaerobic conditions and without agitation. Again, in the presence of bacterial contaminants, the yeast yield was significantly reduced, whereas the addition of phages to the cultures led to reduction in bacterial cell counts and significant recovery in yeast cell counts.

### Ethanol production

**Figure 9:**
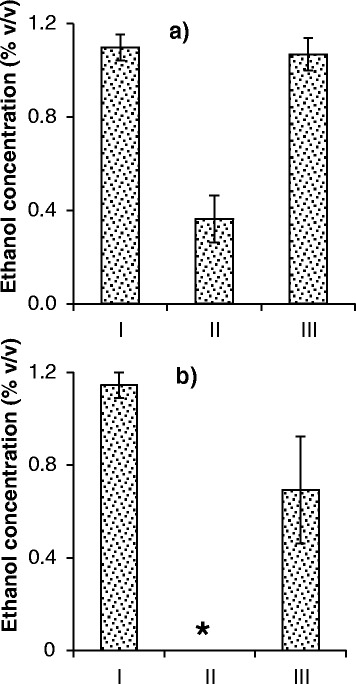
**Ethanol production.** Experiments conducted in MRS medium at pH 6 under **(a)** aerobic and **(b)** anaerobic conditions. Data are reported for cases where yeasts were grown alone (I); in the presence of bacteria at high inoculation level (II); and in the presence of bacteria at high inoculation level and phages B1 and B2 (III). Measurements for aerobic conditions were taken at 24 hours and for anaerobic conditions at 65 hours of fermentation. *Value was below the detection limit of 0.1% ethanol. Error bars indicate the standard deviation of three experiments.

Although ethanol production is generally considered to correlate with yeast growth [34], it is important to evaluate if and how ethanol is affected by the presence of bacteria and phages. Ethanol concentration was measured from experiments performed in MRS medium at pH 6 under aerobic and anaerobic conditions. In preliminary experiments, the production of ethanol by axenic yeast cultures under aerobic conditions was found to reach the highest level at 24 hours, whereas the highest production under anaerobic conditions occurred at 65 hours (data not shown). Figure 9 shows the results for ethanol production (% v/v) at these respective optima. The presence of bacterial contaminants significantly reduced ethanol production to less than 0.4% (v/v) under aerobic conditions and below the detection limit under anaerobic conditions. In both cases, the presence of phages allowed significant recovery in ethanol production, even reaching a statistically equal concentration to that of axenic cultures under aerobic conditions.

## Discussion

LAB, besides being the major group of contaminants in bioethanol fermentation, are commonly used as starter cultures for the production of dairy, meat, vegetable, and probiotic products [35]. Ironically, LAB fermentation faces the constant deleterious issue of contamination by phages [36]. According to Watanabe *et al*. [37], controlling phage contamination is even more difficult than controlling bacterial contamination. The goal of the present research was to take advantage of the spread of phage infection in LAB fermentation processes to reduce contamination and improve biofuel yield in yeast fermentation processes.

### Impact of pH on cultures and infections under aerobic conditions

pH is a parameter with major influence on the growth and productivity of organisms. Reduction of the pH of mash feedstock is a common measure to control contamination; however, because pH values lower than the optimal range for *S. cerevisiae* (pH 5.0 to 5.5) are often used, this can also have a negative impact by affecting both the yield of yeast cells and the ethanol production [7,38,39].

The industrial yeast used in the present study grew similarly over a wide range of pH levels (Figure 1). This pattern reflects the ability of commercial strains to grow efficiently in a pH range from 3.5 to 6 [40]. However, it must be noted that the experiment was carried out aerobically. Under aerobic conditions, *S. cerevisiae* is able to produce 28 molecules of ATP per molecule of glucose, whereas only 2 molecules are produced under anaerobic fermentation [40,41]. Because yeasts waste ATP molecules to pump dissociated hydrogen out of the cell [42], it can be inferred that aerobic conditions allow the yeast to produce enough energy to exclude the H^+^ ions and grow similarly under all pH conditions tested.

The oscillation in optical density observed at approximately 24 hours of fermentation (Figure 1) is likely to be caused by secondary consumption of ethanol produced through the Crabtree effect, which implies that some ethanol is produced under aerobic conditions [43]. It has been reported that, in some cases, *S. cerevisiae* can metabolize this ethanol once all the glucose has been depleted [44,45]. This is consistent with the ensuing increase in optical density observed after 24 hours. In fact, oscillation in yeast development is well known in bioethanol industries.

The pattern for *L. plantarum* growth was different, as expected (Figure 2). Bacteria can use various mechanisms to survive in a medium at non-optimal pH; these vary from exchanging ions for protons with an antiport transport system to synthesizing a variety of proteins [46]. Such mechanisms require energy and probably affect cell yield.

The infectivity of phages was shown to be affected by pH conditions in the medium (Figure 3; Figure 4). This phenomenon was more drastic for phage B1 infections (Figure 3a; Figure 4a), whose infectivity was radically lower at pH values other than pH 6. By contrast, phage B2 was effective over a broader pH range, from 3.5 to 6 (Figure 3b; Figure 4b), with the highest infectivity within the range of pH 4 to 5. For the cocktail of the two phages (Figure 3c; Figure 4c), the infectivity was compounded, remaining high at pH levels ranging from 4 to 6.

Although further investigation is needed to identify the actual infection mechanisms affected by pH in this system, the results from a study conducted by Briggiler Marcó *et al.* [47] on the influence of physiological and environmental factors on the development of phages B1 and B2 can provide clues to the phenomena observed in the present study. Briggiler Marcó *et al.* showed a major reduction in phage B1 infectivity at pH levels lower than 4, due to phage inactivation, whereas in the present study a sharp decrease was observed at pH 5 and below (Figure 3a; Figure 4a). The most probable explanation for the discrepancy in the pH values causing the drastic reduction in infectivity is the difference in experimental conditions (medium and temperature). Reduction in phage infectivity can be caused by changes either in the adsorption process or in the physiological state of the host. Since Briggiler Marcó *et al.* did not notice any reduction in adsorption at pH 5 to pH 7, it could be inferred that change in host development is the most plausible cause for the loss of infectivity. However, it is important to note that ion content and phage stability, which are both factors that influence phage adsorption, differ for different media. The results for phage B2 (Figure 3b; Figure 4b) are in closer agreement with those of Briggiler Marcó *et al.* The main difference is the reduction in infectivity at pH 6 and pH 7 observed in the present study. The former investigation did not find any alteration in infectivity or adsorption over this pH range. Again, changes in adsorption or bacterial physiological state may account for this difference in infectivity, due to the experimental differences in temperature and medium.

### Population dynamics of yeasts, bacteria, and phages B1 and B2

The population dynamics study was conducted in MRS medium at pH 6 under different conditions of aeration, agitation, and bacterial inoculation load (Figure 5; Figure 6; Figure 7; Figure 8). MRS medium [48] was chosen, as it is an appropriate medium for the growth of lactobacillus and for the infection by phages. Yeasts also thrived in this medium. In addition, MRS is a complex medium and thus more closely resembles the industrial mashes used in yeast fermentation for biofuel production, compared with some other synthetic media. Despite this, MRS medium differs from mashes or molasses in several aspects, including sugar content and particulate matter, which could affect infectivity

Yeast cultures growing aerobically in the presence of bacteria at low inoculation load (10^5^ cells/ml) showed a small and statistically non-significant decrease in cell yield in comparison with the axenic culture (Figure 6a, cases I and II). At a bacterial inoculation load of 10^7^ cells/ml, the impact of the presence of contaminants was more obvious, and the decrease in yeast cell counts was significant (Figure 5a; Figure 6a, cases I and III). With both low and high bacterial inoculation loads, introducing a cocktail of phages at an initial multiplicity of infection (MOI) of 0.1 led to significant reductions in bacterial cell counts (more than 99%) (Figure 5c; Figure 6b), and yeast cell counts reached the same levels as axenic yeast cultures (Figure 5a; Figure 6a, cases I, IV and V).

Under anaerobic conditions, bacterial contamination at high inoculation levels also led to significant reduction in yeast yield (Figure 8). The addition of phages lowered bacterial cell counts by more than 65%, and allowed yeast yield to reach 63% of the values for axenic cultures. It should be noted that yeast cell counts in anaerobic experiments were lower than those in aerobic cultures. This phenomenon was observed in all cases: in axenic cultures, in the presence of bacteria, and in the presence of bacteria and phages. Limited agitation and, consequently, limited access to nutrients affected yeast growth in anaerobic cultures. Likewise, phages virulence was reduced in anaerobic experiments. The lack of aeration and/or agitation could have contributed to this phenomenon [49–51]. Lower aeration led to slower bacterial growth and, in turn, to lower burst size (the number of phages released per infected bacterium). In the case of agitation, mass transfer limitation impeded phage adsorption and propagation. Despite this reduction in infectivity under the anaerobic conditions tested, the presence of phages greatly and positively affected yeast cell yield (Figure 8).

### Ethanol production

The production of biofuel by yeasts was also evaluated. Under aerobic conditions, a major reduction (67%) in ethanol concentration was observed when bacteria were competing with yeasts (Figure 9a). When phages were added to the system, ethanol production reached equivalent levels to when yeasts were growing alone. As previously discussed, measurements were taken at 24 hours of fermentation. After this time, the glucose was depleted and the yeast started to consume ethanol.

Similar results were obtained under anaerobic conditions (Figure 9b). No ethanol was detected in the presence of contaminants. The addition of phages allowed considerable recovery of ethanol production (60% of the axenic control). Similar to the yeast cell counts under anaerobic conditions (Figure 8), ethanol concentration in the presence of contaminants and phages remained lower than that in the axenic yeast cultures. This phenomenon reinforces the high correlation existent between yeast cell yield and ethanol production. The explanations for this lower recovery of ethanol production under anaerobic condition are the same as for the yeast cell yield.

The average ethanol yield in these experiments did not surpass 1.2% (v/v). Although this is lower than industrial production levels, it falls within the expected range based on the comparatively low amount of glucose present in the medium used (around 20 g/l or 0.11 mol/l). As the reaction converts one molecule of glucose into two molecules of ethanol, full conversion would result in 0.22 mol/l of ethanol. Approximately 10% of the substrate is used by the yeast for metabolism and multiplication, hence a conversion to ethanol of 90% can be expected [34]. This corresponds to a production of 0.2 mol/l, or 1.17% (v/v) of ethanol, in accordance with the experimental results obtained.

### Feasibility of using phage cocktails as antimicrobial agents in yeast fermentation

These results show the efficacy of using even a simple phage cocktail in an array of conditions. The fact that bacterial cultures inoculated at 10^7^ cells/ml – two orders of magnitude greater than initial contamination levels normally observed in industrial context – were neutralized by phages to such an extent that they did not or only minimally impeded yeast counts and ethanol yields shows the potential of this strategy.

However, questions may be raised regarding the feasibility of using phages as antimicrobial agents in fermentation processes. Five main arguments should be addressed: 1) phage specificity versus diversity of contaminant strains, 2) potential rise of bacterial resistance, 3) range of conditions under which phages can be used, 4) concerns related to the presence of phages in co-products, and 5) cost of production/addition of phage compared to antibiotics. Undoubtedly, more research is needed to address these points fully, but a few insights can be considered currently. 1) Although the variety of contaminants in ethanol fermentation is extensive, it is important to note that the investigations conducted on the identification of these bacteria had a significant number of species in common [10–14]. Moreover, the majority of these contaminants are LAB, hence it can be expected that a single cocktail of phages developed against several LAB species would be effective in a variety of industries in different locations. 2) The emergence of resistance against phages, as against antibiotics, may occur. However, a substantial advantage of phages is that they have the capacity and need to not only multiply rapidly, but also evolve in order to overcome host resistance [52]. In addition, in order to impede resistance, an effective cocktail should be composed of multiple phages against the same bacterial strains, as in the present study. 3) Certainly, more investigation into the optimal conditions for phage application should be carried out to provide comprehensive knowledge on this topic; however the variety of assessments conducted in the present study (including pH, aeration, and agitation) is a starting point for similar studies. Moreover, different phages will exhibit different properties under different ranges of conditions. 4) The presence of active phages in co-products is unlikely, mostly because downstream processes, especially distillation and drying of the distillers’ grains, are sufficient to deactivate or destroy the phages. Even so, the natural ubiquity of phages renders their unlikely presence in by-products less of an issue than with, for example, more potent antibiotics. That being said, the release of large amounts of untreated, active phages in the environment or in the diet of animals could have unexpected impacts on ecosystems and should therefore be treated with caution. 5) Although a complete comparative economic study of the use of phages in lieu of antibiotics in yeast fermentations is yet to be performed, it can be assumed, based on the low costs of production, that phages will be economically appealing. In contrast to antibiotic production, phages are easily amplified and reach high titers rapidly. Moreover, the purification and concentration of phage stocks are typically less costly than those of antibiotics.

Lastly, while the medium used here was not a common industrial substrate (molasses or hydrolyzate), the present study, which is the first investigation of population dynamics for yeast/bacteria/phage systems, is still of relevance to the industrial context. The selection of the tested medium (MRS) was based on the facts that it is a complex medium and that it favors the growth of the bacterial contaminant, and can thus be seen as an extreme case of contamination propagation. These results are encouraging for further studies investigating the application of phages for controlling contaminants in industrial media.

## Conclusions

The present work demonstrated that phages show great potential as antibacterial agents for use in industrial yeast fermentation processes. The use of a simple phage cocktail significantly reduced the impact of *L. plantarum* contamination on the yeast and ethanol yields of a robust commercial *S. cerevisiae* strain. Yeast yield and ethanol production were found to be equal to those of axenic yeast cultures grown under aerobic conditions, and slightly lower under anaerobic conditions without agitation.

## Methods

### Microorganisms and culture media

The organisms utilized in this study were the yeast *Saccharomyces cerevisiae* Superstart™ (Lallemand Biofuels and Distilled Spirits, Montreal, Canada), the bacterium *Lactobacillus plantarum* ATCC® 8014™ and the bacteriophages *Lactobacillus plantarum* ATCC® 8014-B1™ (phage B1) and *Lactobacillus plantarum* ATCC® 8014-B2™ (phage B2).

MRS medium (BD Difco™ lactobacilli broth, Sparks, MD, USA) and M9 minimal medium (7.54 g/l NaH_2_PO_4_, 3.33 g/l KH_2_PO_4_, 1.11 g/l NH_4_Cl, 0.56 g/l NaCl, 0.546 g/l MgSO_4_ · 7H_2_O, 0.016 g/l CaCl_2_ · 2H_2_O, 1.11 g/l of yeast extract, and 2 g/l of glucose) were used for the study. The pH of the media was adjusted to the desired value (from pH 3 to pH 7) with solutions of 1 N HCl and 1 M NaOH.

Bottom and soft agars were prepared by the addition of 1.5% and 0.75% agar (BD Difco, Sparks, MD, USA) in MRS broth, respectively. Bottom agar was immediately poured into petri dishes, while soft agar was kept in a water bath at 60°C prior to cell addition and pouring.

### Cells growth measurements

Cell growth (yeasts and bacteria) was monitored by measuring optical density at 600 nm (OD_600_) using a spectrophotometer (Ultrospec 50; Biochrom, Cambridge, UK); by counting the number of cells in a counting chamber (Improved Neubauer Brightline; Hausser Scientific, Horsham, PA, USA) and a microscope (DMRXA2; Leica Mycrosystems, Heerbrugg, Switzerland) equipped with a digital camera (Retiga EX; QImaging, Surrey, BC, USA); and by the plate count dilution method [53] ,with colony-forming units (CFU) counted on bottom agar incubated overnight at 30°C.

### Phage titer

Phage titer measurements were performed using the agar layering technique [54]. For this, 10 μl (or 2 μl for phage B2) of a bacterial host overnight culture were added to 3 ml of MRS soft agar. The mixture was then poured onto a layer of bottom agar and allowed to solidify for 5 minutes. Dilutions of phage samples were placed on the soft agar, dried, and incubated overnight at 30°C (or 37°C for phage B2). The titer, in plaque forming units (PFU)/mL, was calculated by counting plaques of phages after incubation.

### Aerobic and anaerobic conditions

All aerobic experiments were conducted in 125 ml shake flasks capped with foam stoppers, which were incubated at 30°C with shaking at 150 rpm in a rotary incubator shaker (Ecotron; Infors HT, Bottmingen, Switzerland).

Anaerobic experiments were carried out without agitation in culture bottles, with a capacity of 130 mL, filled with 120 ml of medium. The bottles were sealed to avoid gaseous exchange with the surroundings. Because some oxygen was initially present in the medium and in the headspace of the culture bottles, the conditions were not strictly anaerobic; however, anaerobic conditions were achieved once the dissolved oxygen was consumed by the organisms. These anaerobic experiments simulated typical fermentation conditions encountered in industry.

### Impact of pH on cultures and infections under aerobic conditions

All experiments to evaluate pH effects on organisms were performed aerobically at 30°C with shaking at 150 rpm. OD_600_ measurements were taken periodically.

For experiments with yeast, shake flasks with 25 ml of M9 medium at pH 3, 3.5, 4, 4.5, 5, or 6 were used. The flasks were inoculated with 100 μl of an exponentially growing culture. Two replicates were performed for each experiment.

For the bacteria, M9 at pH 3.5, 4, 4.5, 5, 6, or 7 was used. For these experiments, 20 ml of medium were added to shake flasks and were inoculated with 200 μl of exponentially growing cultures diluted to 10^7^ CFU/mL. Three replicates were performed for each experiment.

Tests involving phages B1 and B2 were carried out in shake flasks with 25 ml of M9 at pH 3.5, 4, 4.5, 5, 6, or 7. Exponentially growing bacteria diluted to 10^7^ CFU/ml were mixed in a microcentrifuge tube with phages at an MOI of 0.1, followed by adsorption (resting) for 5 minutes before 250 μl of the mixture were added to the shake flasks. One replicate was performed for each experiment.

### Inoculation loads for population dynamics studies

Cultures of yeasts and bacteria in the late exponential phase and phage stocks were used as inocula in the experiments. In all cases, the cultures were added to shake flasks to initial concentrations of 10^4^ cells/ml for yeasts, and 10^5^ or 10^7^ cells/ml for bacteria (low and high contamination levels, respectively). Phages were added to obtain an MOI of 0.1. The inoculation load of yeasts followed the recommendation of the yeast manufacturer. The lowest bacterial inoculation level (10^5^cells/mL) simulates typical contamination concentrations found in industry at the beginning of fermentations [12–14], whereas the highest bacterial inoculation level exemplifies an extreme case of contamination. The MOI of 0.1 for phage infections was maintained for single phage strains (B1 or B2) and for the cocktail of these phages (B1 at MOI of 0.05 and B2 at MOI of 0.05, combined to give MOI of 0.1).

### Population dynamics of yeasts, bacteria, and phages B1 and B2

Population dynamics studies were performed in MRS medium at pH 6 under aerobic and anaerobic conditions. The aerobic experiments were conducted in shake flasks containing 25 ml of medium, and were divided in two, one to have a single measurement after 20 hours of fermentation and the other to have periodic measurements throughout 60 hours of fermentation. High or low bacterial inoculations were added, as appropriate, and the experiments were conducted in triplicate and duplicate, respectively. All anaerobic experiments were carried out in triplicate.

### Ethanol production

In experiments without phages, 1 ml culture samples were transferred into microcentrifuge tubes and centrifuged (5424R centrifuge, FA-45-24-11 rotor; Eppendorf Hamburg, Germany) for 1 minute at 14,000 × g and 25°C. When samples contained phages, centrifugation was carried for 90 minutes at 20,000 × g and 25°C to remove phages prior to analysis. Samples were filtered (0.2 μm; Fisher Scientific, Windsor, ON, Canada) and the ethanol content was measured by high performance liquid chromatography (Agilent Technologies 1200 series, SupelcoGel Pb carbohydrate column; 7.8 mm internal diameter and 30 cm length, with guard column and refractive index detector). Sample injection volume for measurement was 10 μl, and the elution flow rate was 0.5 ml/min with deionized sterile water (MilliQ; MilliPore, Etobicoke, ON, Canada). Measurements were performed in triplicate.

### Statistical analysis

Variance and *t*-test analyses, with 95% confidence level (*P* < 0.05), were conducted for comparison of averages of triplicates.

## Abbreviations

ATCC: American Type Culture Collection
CFU: Colony forming units
LAB: Lactic acid bacteria
MOI: Multiplicity of infection
PFU: Plaque forming units
